# The impact of night shift stress and sleep disturbance on nurses quality of life: case in Palestine Red Crescent and Al-Ahli Hospital

**DOI:** 10.1186/s12912-023-01673-3

**Published:** 2024-01-08

**Authors:** Jebril AL-hrinat, Abdullah M. Al-Ansi, Aseel Hendi, Ghadeer Adwan, Manar Hazaimeh

**Affiliations:** 1 Faculty of Education, Arab American University, Hebron, Palestine; 2 Faculty of Education, Thamar University, Thamar, Yemen; 3 Industrial Engineering Department, the Hashemite University, Zarqa, Jordan; 4 Faculty of Education, Islamic University of Gaza, Gaza, Palestine; 5 Department of Counseling and Educational Psychology, Yarmouk University, Irbid, Jordan

**Keywords:** Night shift stress, Nurses, Quality of life, Sleep disturbances

## Abstract

**Background:**

Nurses play a vital role in providing round-the-clock care to patients, but the challenges associated with working night shifts can have significant implications for their well-being and quality of life.

**Methods:**

This cross-sectional study aimed to investigate the impact of night shift stress and sleep disturbance on the quality of life among nurses working in Palestine Red Crescent Society and Al-Ahli Hospital. Convenience sampling was used to recruit 189 full-time registered nurses with at least one year of job experience. The participants completed a questionnaire assessing night shift stress, sleep disturbance, and quality of life. Descriptive statistics, correlation analysis, and path analysis were conducted to analyze the data.

**Results:**

The results reveal that quality of life has positive and low relationship with both night shift stress and sleep disturbances. Results also reveal that night shift stress had a direct negative impact on the quality of life of nurses. Sleep disturbance was found to mediate the relationship between night shift stress and quality of life, indicating that higher levels of night shift stress were associated with increased sleep disturbance, which, in turn, led to poorer quality of life outcomes.

**Conclusion:**

These findings highlight the importance of addressing night shift stress and sleep disturbance among nurses to enhance their well-being and improve the quality of care provided to patients. In conclusion, this study contributes to the existing literature by demonstrating the detrimental effects of night shift stress and sleep disturbance on the quality of life of nurses. It emphasizes the importance of implementing interventions and creating supportive work environments that address the unique challenges faced by nurses working night shifts.

## Introduction

Nurses play a critical role in providing round-the-clock care and support in the healthcare system [1]. However, the demands of working night shifts can result in significant stress and sleep disturbances, impacting the overall well-being and quality of life of nurses [2]. The problem of night-shift is particularly relevant in developing countries like Palestine, where nurses often face challenging working conditions and limited resources. The Palestine Red Crescent Society and Al-Ahli Hospital are prominent healthcare institutions in Palestine that employ a substantial number of nurses. It is crucial to comprehend the effects of night shift stress and sleep disturbance on the quality of life of nurses in these establishments to develop effective interventions that enhance their well-being and ultimately improve patient outcomes [3]. This study aims to investigate the impact of night shift stress and sleep disturbance on the quality of life among nurses working at the Palestine Red Crescent Society and Al-Ahli Hospital. The findings from this study will inform policies and practices aimed at supporting nurses’ mental and physical health and enhancing the quality of care provided to patients.

Night shift work can disrupt sleep patterns and induce stress [4–8]. It can disturb the natural rhythms of cortisol and melatonin, which are closely tied to sleep quality [4]. Insufficient sleep is a common complaint among night-shift workers, leading to difficulties in daytime sleep after night shifts [4]. Night-shift workers face an increased risk of sleep disturbances, and longer shift durations can contribute to occupational injuries [6]. Shift workers also exhibit a higher prevalence of insomnia and mental disorders compared to non-night-shift workers [6]. Shift work sleep disorder (SWSD) is a prevalent sleep disorder affecting individuals who work night shifts, early morning shifts, or rotating shifts. Symptoms of SWSD include difficulties falling asleep, excessive sleepiness, reduced concentration, headaches, and lack of energy [5]. To mitigate the effects of night shift work, it is recommended to follow consistent sleep schedules and rituals, even on weekends and days off, and prioritize adequate sleep during rest days while practicing good sleep hygiene by avoiding substances like caffeine, alcohol, and nicotine [5]. Extended work hours, heavy workloads, and emotional demands are significant factors contributing to sleep disturbances among night-shift workers [8]. These findings underscore the importance of adjusting work demands and minimizing extended work durations to alleviate sleep disturbances among night-shift workers [8]. Guidelines for employees and companies are available to manage work stress and promote the health of shift workers [6]. Night-shift workers must prioritize their sleep and mental health to mitigate the negative effects of night shift work.

According to the World Health Organization (WHO), quality of life refers to an individual's perception of their position in life relative to their goals, expectations, standards, and concerns, taking into account the cultural and value systems they belong to [9]. It is a crucial concept in healthcare as it helps assess the impact of health conditions and treatments on a person's life [10]. Night shift work can have adverse effects on both physical and mental health, as well as quality of life. Research indicates that night shift workers experience disruptions in their circadian rhythms, leading to sleep disturbances and increased oxidative stress [11]. Mental health consequences of shift work include depressed mood, anxiety, substance use, cognitive impairments, and even suicidal thoughts [12].

Night shift work has also been associated with cardiovascular disease, obesity, digestive problems, and diabetes [13]. A study revealed that night shift work was linked to lower health-related quality of life, with sleep quality acting as a mediating factor. A study revealed that night shift work was linked to lower health-related quality of life, with sleep quality acting as a mediating factor [13]. It is crucial for night shift workers to prioritize their health and well-being while taking steps to mitigate the negative effects of shift work [14]. Prior studies have shown that sleep disturbances can impact the quality of life and health of nurses [15, 16]. Nurses often work long hours with irregular schedules, which can lead to burn out and affect their professional quality of life [15, 16]. Furthermore, lack of sleep can also have a detrimental impact on the quality and safety of patient care [17]. Therefore, it is essential for healthcare managers to consider implementing interventions that support nurses' sleep and improve their overall health and job performance. Further research is needed to gain a better understanding of the factors contributing to sleep disturbances among nurses and to develop effective interventions to address this issue.

### Conceptual framework

**Fig. 1:**
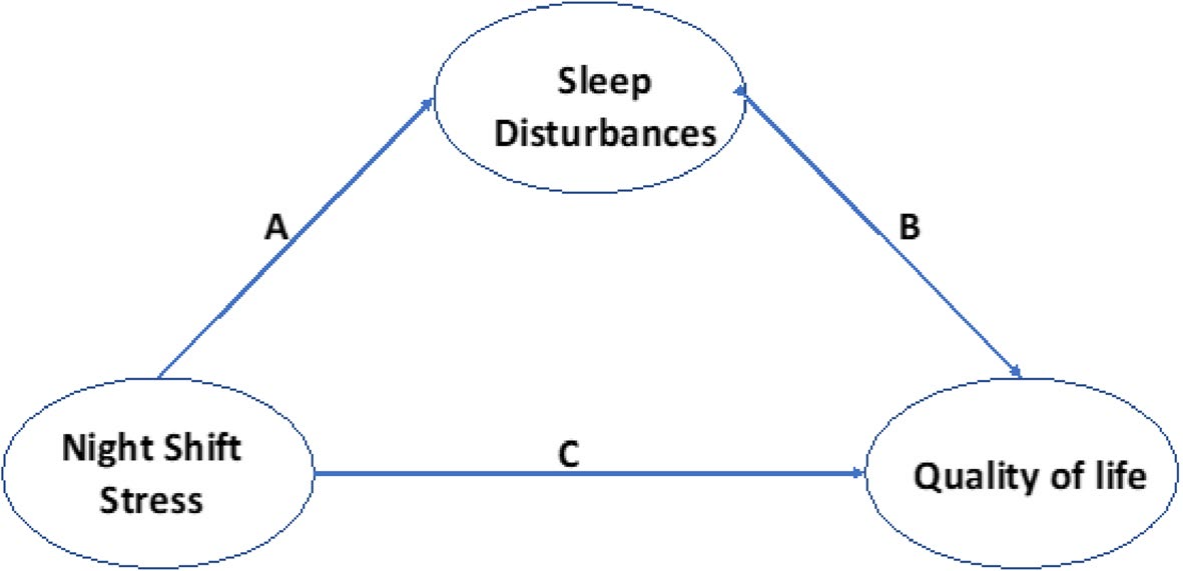
Research framework

This study builds upon Punnett theoretical framework, which asserts that the connection between working conditions and employee health is multifaceted [18]. According to the framework (Fig. 1), health behaviors, including sleep patterns, play a significant role in the relationship between working conditions and employees' health and quality of life. Unfavorable working conditions, such as exposure to night shift stress, can directly impact an employee's physical and mental well-being, as well as their quality of life. Moreover, night shift stress can lead to adverse health behaviors, such as disrupted sleep, which further affect both physical and mental health. Conversely, positive working conditions, such as high levels of control and support, can act as protective factors for an individual's health. However, the mechanisms underlying the relationship between quality of life and night shift stress among nurses have yet to be empirically investigated. Consequently, it is crucial to comprehend the interplay between quality of life, sleep disorders, and night shift stress to develop more effective interventions aimed at enhancing nurses' overall well-being [3].

A theoretical framework proposes that sleep disturbance acts as a mediator between night shift stress and quality of life. This means that the impact of night shift stress on quality of life is not only direct (path c), but also indirect through its effect. The primary objectives of this study are to examine the associations among night shift stress, sleep disturbance, and quality of life, as well as to explore the mediating role of sleep disturbance in the relationship between quality of life and night shift stress among nurses. Drawing from Punnett proposed pathways and existing research findings, the following hypotheses are postulated:

- HA: Night shift stress positively associated with Sleep disturbance among nurses.
- HB: Sleep disturbance negatively associated with quality of life among nurses,
- HC: Sleep disturbance mediates the relationship between night shift stress and quality of life.

## Methods

### Design

This study employed a cross-sectional design to examine the relationship of night shift stress and sleep disturbance with the quality of life of nurses working in Palestine Red Crescent Society and AL-Ahli Hospital.

### Participants

A total of 207 nurses employed at the Palestine Red Crescent Society and Al-Ahli Hospital participated in this study. Convenience sampling was employed to select individuals who met the inclusion criteria, which included being full-time registered nurses with a minimum of one year of job experience, all nurses not (registered nurses) and expressing willingness to participate in the study. 189 nurses have completed questionnaires where 18 of them were excluded form the sample.

### Procedures and data collection

To conduct this study, an online survey questionnaire was employed as the main instrument for data gathering. The development of the questionnaire was informed by an extensive review of relevant literature from diverse sources including Scopus database, Google Scholar, ProQuest, Midline, and ResearchGate. The questionnaire was constructed by formulating a set of questions tailored to the research sample, and its validity was confirmed through expert consultation and subsequent modifications based on their feedback. To ensure the reliability and validity of the questionnaire, the researchers employed Cronbach's alpha, which is a method for assessing internal consistency. The questionnaire of this study was adopted from prior works including: night shift stress [19], sleep disturbance [20, 21], quality of life [20]. These questionnaires were translated to Arabic language and reviewed many times by researchers and both ethics committees from Palestine Red Crescent Society and Al-Ahli Hospital.

### Construct reliability and validity

In the initial run of the measurement model, there were no indicators with poor lower factor loadings (< 0.70). However, considering the potential influence of this issue on the results, the study decided to accept all indicators as most of them exhibited strong factor loadings (> 0.70) [22].

Reliability refers to the consistency and stability of measurement items, ensuring that they produce consistent results across different conditions and consistently over time [23]. Cronbach's Alpha and Composite Reliability (CR) are commonly used to assess reliability, taking into account the various loadings of the measurement items. Higher values of CR or Cronbach's alpha indicate greater reliability. The minimum acceptable threshold for internal consistency is typically set at 0.7. In Table 1, the results of this analysis demonstrate satisfactory reliability, as all values of Cronbach's Alpha and CR exceed 0.7.

**Table 1:** Descriptive statistics, reliability and validity test

**Item**	**FL**	**M**	**SD**	**Cronbach’s alpha**	**CR**	**AVE**
**QoL**						
Q1	0.745	4.01	0.873	0.876	0.892	0.712
Q2	0.823	4.11	0.924			
Q3	0.865	4.19	0.922			
Q4	0.776	4.10	0.978			
Q5	0.876	3.73	0.902			
Q6	0.833	3.85	0.979			
Q7	0.723	4.17	1.010			
Q8	0.765	4.40	0.980			
Q9	0.801	3.89	0.967			
**NSS**						
Q1	0.711	4.10	0.901	0.901	0.889	0.695
Q2	0.723	3.76	0.966			
Q3	0.786	3.97	0.923			
Q4	0.876	4.01	0.990			
Q5	0.781	4.18	0.912			
Q6	0.786	3.87	0.910			
Q7	0.801	4.20	0.911			
Q8	0.771	4.21	0.987			
**SD**						
Q1	0.720	3.40	0.913	0.892	0.917	0.710
Q2	0.821	4.21	0.946			
Q3	0.763	4.19	0.994			
Q4	0.790	3.98	1.017			
Q5	0.813	3.87	0.97			
Q6	0.828	3.92	1.012			
Q7	0.825	3.94	0.888			
Q8	0.785	3.93	0.996			

In addition, Convergent validity is employed to assess the degree of association among multiple metrics or indicators used to develop a model [24]. In the current study, convergent validity was evaluated using Average Variance Extracted (AVE). To meet the acceptable threshold, the AVE value should reach a minimum of 0.50, as suggested by [25], for future analysis. The results indicate that all constructs exceeded the AVE cutoff of 0.50, demonstrating good convergent validity.

### Discriminant Validity

Discriminant validity is an essential concept evaluated using various methods, including the Fornell-Larcker criterion and Heterotrait-Monotrait ratio. The subsequent table, labeled as Table 2, displays the outcomes of these assessments, underscoring their importance in appraising discriminant validity.

**Table 2:** Fornell-Larcker criterion

	QoL	NSS	SD
QoL	0.772		
NSS	0.787	0.789	
SD	0.726	0.767	0.795

### Data analysis

Descriptive statistics were utilized to characterize the study sample, including calculating means and frequencies to describe demographic characteristics. Prior to conducting the modeling, assumptions concerning the normality of outcomes and potential mediators were evaluated. Furthermore, the Harman's single-factor test was implemented to identify any common method bias, and the results indicated that it did not pose a significant threat in this study. The relationships among night shift stress, sleep disturbance, and quality of life were assessed by using Pearson correlation. To examine the mediating role of sleep disturbance between night shift stress and quality of life in nurses, path analysis was employed. Age and gender were predetermined as covariates and were controlled for in all models based on existing evidence. Several fit indices, including RMSEA < 0.08, CFI ≥ 0.95, and SRMR ≤ 0.08 0.19 were employed to evaluate the model's goodness of fit. The significance of the mediation effects was evaluated using non-parametric, bias-corrected 95% bootstrapped confidence intervals (BCI) with 5,000 bootstrap replications [26, 27]. If the 95% BCI did not include zero, the indirect effect was considered statistically significant. All mediation analyses were conducted using the process macro for SPSS version 27. A significance level of *p* < 0.05 was used to determine statistical significance.

## Results

### Characteristics of study subjects

Table 3 presents the general characteristics of the participants in the study, primarily focusing on their age distribution. Majority of participants were female with 73%. In addition, the majority of the nurses fell within the age range of 20 to under 30 years, comprising approximately 71.40% of the sample. The next highest age category was between 30 and under 40 years, accounting for about 25.4% of the participants. Regarding marital status, a significant proportion of the nurses were married, representing 55.60% of the total. Furthermore, the highest percentage of participants possessed a bachelor's degree, with a rate of 71.40%. The average values reported by the nurses for gender was 1.41 and 1.49, respectively.

**Table 3:** Descriptive characteristics for nurses

Demographics		Number	%
Gender	Female	138	73%
	Male	51	27%
Age	20 years to less than 30 years	134	71.40%
	30 years to less than 40 years	48	25.40%
	40 years to less than 50 years	17	3.20%
	50 years and over	0	0.00%
Marital Status	single	84	44.40%
	married	105	55.60%
Educational level	diploma	24	12.70%
	Bachelor's degree	134	71.40%
	Postgraduate	31	15.90%

### Association between night shift stress, sleep disturbance, and quality of life

In the initial analysis, several statistically significant relationships were identified between night shift stress, sleep disturbance, and quality of life in Table 4. Results reveal that quality of life has positive and low relationship with both night shift stress and sleep disturbances. The Table 4 also shows the relationship between night shift stress and sleep disturbance which is also positive and low relationship.

**Table 4:** Correlational among night shift stress, sleep disturbances and quality of life

**Variables**		**Quality of life**	**Night shift Stress**	**Sleep disturbances**
**Quality of life**	**Pearson Correlation**	1	.344**	0.187*
	**Sig. (2-tailed)**		0.000	0.051
	**N**	189	189	189
**Night shift Stress**	**Pearson Correlation**	.344**	1	.353**
	**Sig. (2-tailed)**	0.000		0.000
	**N**	189	189	189
**Sleep disturbances**	**Pearson Correlation**	0.187	.353**	1
	**Sig. (2-tailed)**	0.051*	0.000	
	**N**	189	189	189

** Correlation is significant at the 0.001 level (2-tailed); * Correlation is significant at the 0.01 level (2-tailed)

### Associations between night shift stress, sleep disturbance, and quality of life

Table 5 provides an overview of the direct and indirect effects of night shift stress and sleep disturbance on the quality of life for nurses. These results are illustrated in the three following models.

**Table 5:** Direct and indirect effects analysis

	**R**	**R-sq**	**MSE**	**F**	**df1**	**df2**	**P**
**Model Summary (path A)**	0.6534	0.2789	18.8105	15.413	1	108	0.0002
**Model Summary (path B)**	0.6506	0.4232	18.1763	7.4987	2	107	0.0009
**Model Summary (path C)**	0.6436	0.4142	14.4584	18.108	1	108	0.0002
**Model 1(path A)**							
	coeff	se	t	p	LLCI	ULCI	
**Constant**	35.45	4.11	8.61	0.001	27.29	43.6	
**Night shift stress**	0.38	0.09	3.92	0.002	0.19	0.58	
**Model 2(path B)**							
**Constant**	29.6313	5.2529	5.641	0.001	19.218	40.045	
**Night shift stress**	0.3384	0.1032	3.2781	0.0014	0.1338	0.5431	
**Sleep disturbances**	0.0729	0.0946	0.7703	0.0428	-0.115	0.2604	
**Model 3(path c)**							
**Constant**	32.2144	4.0356	7.9825	0.001	24.22	40.21	
**Night shift stress**	0.3665	0.0964	3.8024	2E-04	0.176	0.558	
**Total effect of X on Y**							
Effect	se	t	p	LLCI	ULCI		
0.3665	0.0964	3.8024	0.0002	0.1755	0.5576		
Direct effect of X on Y							
Effect	se	t	p	LLCI	ULCI		
0.3384	0.1032	3.2781	0.0014	0.1338	0.5431		
**Indirect effect(s) of X on Y:**							
	Effect	BootSE	BootLLCI	BootULCI			
**Sleep Disturbances**	0.0281	0.0422	-0.0466	0.1194			

#### Model A

The first regression analysis reveals that night shift stress significantly and positively predicts sleep disturbances (b = 0.38, s.e. = 4.11, *p < *0.001). This coefficient represents the direct impact of night shift stress on sleep disturbances within the path model.

#### Model B

The second regression analysis demonstrates that both night shift stress (b = 0.3384, s.e. = 0.1032, *p* < 0.0014) and sleep disturbances (b = 0.3384, s.e. = 0.1032, *p* = 0.0014) are significant and positive predictors of quality of life among nurses. These coefficients reflect the direct effects of both sleep disturbances and night shift stress on quality of life within the path model.

#### Model C

The third regression analysis shows that night shift stress is a significant positive predictor of quality of life (b = 0.3665, s.e. = 0.0964, *p* < 0.0002). This coefficient represents the direct effect of night shift stress on quality of life within the path model.

Finally, the unstandardized indirect effect (0.0281) of sleep disturbances is calculated as the product of paths a (0.38) and b (0.0729) from the previous regression models. This indirect effect is tested using bootstrap standard errors and confidence intervals. The null hypothesis assumes that the population indirect effect is zero, while the alternative hypothesis suggests a non-zero population indirect effect. If the lower and upper bounds of the confidence interval (typically set at 95%) contain zero, the null hypothesis is maintained. If zero falls outside the interval, the null hypothesis is rejected. In this case, the null hypothesis is rejected.

The total effect of night shift stress on quality of life is computed as the sum of the direct effect (0.3384) and indirect effect (0.0281), resulting in a total effect of 0.3665. Since zero (the null) does not fall within the lower and upper bounds of the 95% confidence interval, it can be inferred that the total effect of night shift stress on quality of life is significantly different from zero.

## Discussion

The primary objective of this study was to examine the direct impact of night shift stress on sleep disturbance and quality of life among nurses, as well as investigate the mediating role of sleep disturbance in the relationship. Our findings align with previous studies, indicating that nurses working night shifts and experiencing work-related pressure have a significant negative impact on their quality of life and health [12–14]. These results are consistent with previous research that has demonstrated a direct association between night shift stress and sleep disturbances, including insomnia [4–8].

Night shift work stress has been identified as a significant factor contributing to decreased quality of life among nurses [13, 14]. The disruption of the circadian rhythm caused by working at night leads to physiological and psychological disturbances, resulting in fatigue, reduced job satisfaction, and increased stress levels among nurses. These stressors can have long-term implications, including the development of chronic diseases and mood disorders. It is crucial to address night shift work stress and create supportive work environments to promote the well-being of nurses.

Sleep disorders are prevalent among nurses who work night shifts, with common complaints including insomnia, circadian rhythm disorders, and excessive daytime sleepiness [4–8].

These sleep disturbances have adverse effects on cognitive functioning, physical health, and social well-being. Nurses may experience difficulties in concentration, memory recall, and decision-making, which can impact the quality of patient care. The increased risk of obesity, diabetes, and cardiovascular diseases associated with sleep disorders further underscores the need for intervention [28].

The implications of night shift work stress and sleep disorders extend beyond individual nurses and can impact the overall quality of patient care [14, 17]. Fatigue and impaired cognitive functioning can compromise nurses' ability to provide optimal care, potentially leading to medical errors and compromised patient safety [29]. It is therefore essential to prioritize the well-being of nurses and address the challenges they face [15, 16].

### Implications for practice

#### Physical health implications

Night work pressure and disrupted sleep patterns can have severe consequences on the physical health of nurses. Irregular sleep schedules and prolonged night shifts can disrupt the body's natural circadian rhythm, leading to sleep disorders such as insomnia, fatigue, and decreased immune function. These health issues can further result in increased susceptibility to illnesses, chronic fatigue, and even long-term health problems such as cardiovascular diseases [30].

#### Mental health implications

The combination of night work pressure and sleep disturbances can have a significant impact on the mental health of nurses. The constant adjustment between day and night shifts can disrupt their social life, leading to feelings of isolation, stress, and depression. Additionally, the pressure of working during nighttime hours, dealing with emergencies, and making critical decisions can contribute to heightened anxiety levels, burnout, and emotional exhaustion. These mental health implications can affect the nurse's overall job satisfaction and their ability to provide optimal patient care [13].

#### Impaired cognitive function

Lack of adequate sleep and the strain of night work pressure can impair cognitive function among nurses. Sleep deprivation can lead to difficulties in concentration, memory lapses, and reduced cognitive processing speed. This can impact decision-making abilities and the nurse's overall performance, potentially compromising patient safety. In high-stress environments like hospitals, these cognitive impairments can have serious consequences, increasing the risk of medical errors and compromising the quality of care provided [31].

#### Work-life balance challenges

The demanding nature of night shifts and the resulting impact on nurses' physical and mental well-being can create significant challenges in maintaining a healthy work-life balance. Sleep deprivation and disrupted social schedules can lead to strained relationships with family and friends, limited participation in social activities, and a decreased overall quality of life. The inability to maintain a proper work-life balance can contribute to increased stress levels and feelings of professional dissatisfaction, potentially leading to burnout and a higher turnover rate within the nursing profession [32].

#### Patient care and safety

Perhaps the most critical implication of night work pressure and sleep strikes is the potential compromise of patient care and safety. Sleep-deprived nurses may experience decreased vigilance, impaired critical thinking, and slower response times. These factors can increase the likelihood of medical errors, compromise decision-making, and decreased overall patient satisfaction. Maintaining an optimal level of alertness and attentiveness is essential in healthcare settings, and addressing the challenges associated with night work pressure and sleep strikes is crucial for maintaining the highest standards of patient care [33].

Finally, the implications of night work pressure and sleep strikes on the quality of life for nurses in hospitals are significant and far-reaching. It is crucial for healthcare institutions to recognize and address these issues by implementing policies and practices that prioritize the well-being of nurses, such as adequate breaks, flexible scheduling, and providing support systems to mitigate the negative effects. By taking proactive measures, hospitals can ensure the physical and mental well-being of their nursing staff, enhance patient safety, and improve the overall quality of care.

### Limitations and future directions

#### Limitations

1. The study employed a cross-sectional design, which limits our ability to establish causal relationships between night shift stress, sleep disturbance, and quality of life. Longitudinal studies would be valuable in examining the temporal nature of these associations.
2. The study relied on self-report measures, which are subject to response biases and may not capture the full complexity of the constructs under investigation. Future studies could benefit from incorporating objective measures, such as actigraphy or physiological markers, to provide a more comprehensive assessment.
3. Convenience sampling was utilized, which may introduce sampling bias and limit the generalizability of the findings to other populations of nurses.
4. The study focused on nurses working in specific healthcare institutions in Palestine, which may limit the generalizability of the results to nurses in other settings or countries.

#### Future directions

1. Further research should explore the potential underlying mechanisms that link night shift stress, sleep disturbance, and quality of life among nurses. Investigating factors such as coping strategies, social support, and individual resilience could enhance our understanding of the complex relationships involved.
2. Longitudinal studies are needed to examine the long-term effects of night shift work on the health and well-being of nurses. This would enable the identification of potential cumulative effects and the development of targeted interventions.
3. Comparative studies could be conducted to examine the differences in night shift stress, sleep disturbance, and quality of life among nurses working in various healthcare settings or across different countries.
4. Intervention studies are warranted to evaluate the effectiveness of interventions targeting night shift stress and sleep disturbance among nurses. Such interventions could include workplace interventions, educational programs, and individual coping strategies.
5. Exploring the role of organizational factors, such as staffing levels, shift scheduling practices, and workplace support, would provide valuable insights into how the work environment can be optimized to mitigate the negative impact of night shift work on nurses' well-being.
6. Additionally, investigating the impact of sleep disorders and night shift work on specific patient outcomes, such as patient safety, satisfaction, and healthcare quality, would contribute to the broader understanding of the implications of these factors in the healthcare setting.

## Conclusion

In conclusion, this study examined the impact of night shift stress and sleep disturbance on the quality of life among nurses working in Palestine Red Crescent Society and Al-Ahli Hospital. The findings of this study provide valuable insights into the challenges faced by nurses working night shifts and their implications for well-being and quality of life. The results demonstrated that night shift stress has a direct negative impact on the quality of life of nurses. Additionally, sleep disturbance was identified as a significant mediator in the relationship between night shift stress and quality of life. Higher levels of night shift stress were associated with increased sleep disturbance, which, in turn, led to poorer quality of life outcomes. These findings highlight the importance of recognizing and addressing the unique challenges faced by nurses working night shifts. It is crucial for healthcare organizations to implement strategies and interventions that aim to reduce night shift stress and improve sleep quality among nurses. This may include creating supportive work environments, implementing adequate staffing levels, offering resources for coping with night shift challenges, and providing education and training on sleep hygiene and self-care practices.

By prioritizing the well-being of nurses, healthcare organizations can not only enhance the quality of life of their nursing workforce but also improve patient outcomes. Nurses who experience reduced stress and better sleep are likely to provide higher-quality care, exhibit improved job satisfaction, and experience fewer instances of burnout. However, it is important to acknowledge the limitations of this study, including its cross-sectional design and reliance on self-report measures. Further research employing longitudinal designs and objective measures would help establish causal relationships and provide a more comprehensive understanding of the complex interplay between night shift stress, sleep disturbance, and quality of life among nurses. Overall, this study contributes to the growing body of knowledge on the challenges faced by nurses working night shifts and their impact on well-being. It emphasizes the need for organizational support, interventions, and policies that promote the health and well-being of nurses in order to create a sustainable and thriving nursing workforce.

## Data Availability

The datasets used and/or analyzed during the current study available from the corresponding author on reasonable request.
